# The Effect of Low-Haze Diffuse Glass on Greenhouse Tomato and Bell Pepper Production and Light Distribution Properties

**DOI:** 10.3390/plants9070806

**Published:** 2020-06-27

**Authors:** Kristof Holsteens, Rob Moerkens, Bram Van de Poel, Wendy Vanlommel

**Affiliations:** 1 Division of Crop Biotechnics, Department of Biosystems, University of Leuven, Willem de Croylaan 42, B-3001 Leuven, Belgium; kristof.holsteens@kuleuven.be (K.H.); bram.vandepoel@kuleuven.be (B.V.d.P.); 2 Fruit vegetable research, Research Centre Hoogstraten, Voort 71, B-2328 Hoogstraten, Belgium; Rob.Moerkens@biobestgroup.com

**Keywords:** greenhouse coverings, diffuse glass, vertical light distribution, haze, yield, *Solanum lycopersicum*, *Capsicum annuum*

## Abstract

Diffuse greenhouse glass can increase the production and growth of several crops, by scattering the incoming direct sunlight, which results in a better and more homogeneous light distribution in the crop canopy. Tomato and bell pepper growers in Belgium tend to install low-haze diffuse glass with a double anti-reflection (AR) coating. These glass types have a limited diffuse effect but have a higher light transmission compared to standard float glass. Therefore, tomato growers often increase stem density to maximize light interception. However, a denser crop could counteract the positive effects of diffuse glass on the vertical light distribution. In this study, the effect of low-haze diffuse glass with an AR coating was evaluated for different cropping densities for tomato and bell pepper taking into account the vertical light distribution throughout the crop canopy. Tomato plants with two stem densities (3.33 and 3.75 stems.m^−2^) and bell pepper plants (with only one stem density of 7.1 stems.m^−2^) were evaluated in a greenhouse compartment with diffuse and reference float glass during a full growing season. For tomato, a significant production increase of 7.5% was observed under diffuse glass during the second half of the growing season but only for the low stem density. The benefit of diffuse glass appears most relevant during sunny clear skies and on the sun-side-facing rows of the crop. For bell pepper, no significant production increases were noted between regular float or diffuse glass, because a bell pepper crop is typically covered with thermal screens to prevent sunburn on the fruits during sunny days. The vertical light distribution and the usefulness of AR-coated diffuse glass depends on the crop type and should be optimized accordingly by altering the stem density, leaf pruning strategy, row orientation, or crop variety.

## 1. Introduction

Sunlight is one of the most important environmental factors that influences plant growth and eventually yield in commercial greenhouses. Besides light intensity, the directional light distribution throughout the canopy also plays an important role [1]. A crop perceives sunlight heterogeneously because it is mainly composed of direct light that arrives in a straight line, of which the intensity exponentially decreases throughout the canopy [2]. Consequently, at a certain canopy depth, some leaves are heavily shaded and thus do not perceive direct light. However, sunlight is also composed of a small fraction of diffuse light that arises from the scattering of direct light by particles in the atmosphere [3]. This scattered or diffuse light results in a more uniform light distribution that penetrates deeper into the crop canopy [1,4]. However, at a single leaf level, the net photosynthesis under diffuse light was found to be lower than under direct light [5,6]. This is because leaves fully exposed to high irradiance direct light are morphologically adapted by having a thick mesophyll layer, which is not the case for thinner shaded leaves [7]. In these fully exposed thick leaves, diffuse light has a lower penetration depth, which reduces the net photosynthetic capacity compared to direct light. Shaded leaves, however, do not discriminate between light direction and thus maintain the same photosynthetic capacity for both direct and indirect light [8,9]. Diffuse light creates no shading patterns and has a more homogeneous light distribution throughout the canopy, which will result in a relatively larger leaf area exposed to light (including lower canopy leaves). This effect will result in higher net photosynthesis at the crop level [10,11], leading to higher biomass production [1,5,12,13]. Furthermore, diffuse light might also benefit photosynthetic efficiency due to a lower leaf temperature and less photoinhibition. Altogether, plants can use diffuse light more efficiently compared to direct light [3,13].

On clear days, sunlight is only composed of 15% diffuse light, while on cloudy days, the percentage of diffuse light reaches 95–100% [3]. Plants exposed to more diffuse light in natural ecosystems have shown an enhanced light use efficiency (LUE) and a higher biomass production compared to the same light intensity when given direct light [10,14,15,16,17,18]. The beneficial properties of diffuse light can also be exploited in greenhouse production systems by using diffuse glass. This diffuse glass will scatter direct incoming sunlight and has already been proven useful in horticulture. It was shown that diffuse glass can significantly increase the production of several vegetable crops, such as cucumber [19] and tomato [12], as well as ornamental plants, such as *Chrysanthemum* [19,20], rose [21], and *Anthurium* [22]. The quantity of light scattering trough diffuse glass, and thus the amount of diffuse light it generates, is indicated by the haze factor. The study of Dueck et al. [12] reported an increase in tomato production, caused by an increased fruit weight, varying from 9–11% for a high haze factor (45–71% haze) and 5% for a mid-haze factor (50% haze). 

Nowadays, diffuse glass has become a common practice in modern Belgian and Dutch tomato and bell pepper greenhouses. Although previous studies indicated that a high haze factor results in higher yields, most Belgian tomato and bell pepper growers choose glass types with a lower haze. Because a higher haze implies a lower light transmission, growers prefer a diffuse glass with a low haze factor that allows more direct light to be transmitted. An extra argument for using low-haze glass is the lower cost. Selecting the correct haze factor is a matter of discussion and remains a difficult decision for growers. Another common practice in greenhouse cultivation is the choice of glass with a double anti-reflection (AR) coating, which reduces the loss of light transmission significantly. By choosing an AR coating in combination with low-haze glass, the light transmission is often higher compared to the standard 0% haze glass. In this case, tomato growers often choose to cultivate plants with a higher stem density. This higher stem density results in a denser crop, which will in turn influence the light distribution within the crop canopy. It remains unclear whether the positive effects of diffuse glass are still valid in a denser tomato canopy. On the other hand, northwest European greenhouses typically cultivated bell pepper that is even denser in comparison to tomato. The effect of low-haze glass on the production of bell pepper remains an open question. 

In this paper, we investigated the effect of low haze diffuse glass with a double AR coating on the yield of tomato and bell pepper in a semi-commercial greenhouse. We tested the hypothesis that a denser canopy will benefit under low-haze diffuse glass and that it will increase production. 

## 2. Results

### 2.1. Climate Data

**Figure 1:**
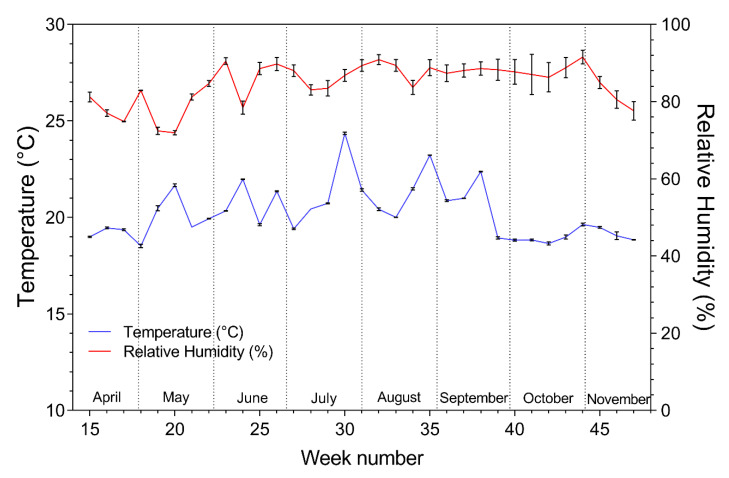
Weekly mean (± SD) temperature and relative humidity in the reference and diffuse tomato compartment.

The daily mean temperature in the tomato greenhouse compartments was 19.62 ± 0.10 °C in the diffuse and 19.68 ± 0.10 °C (mean ± standard error (SE)) in the reference treatment during the total length of the trial. The daily mean relative humidity was 82.6 ± 0.5% in the diffuse and 81.6 ± 0.4% (mean ± SE) in the reference treatment. The weekly mean temperature and relative humidity in the reference and diffuse compartments were equivalent during the total length of the trial, as indicated by the standard deviation (SD) in Figure 1.

**Figure 2:**
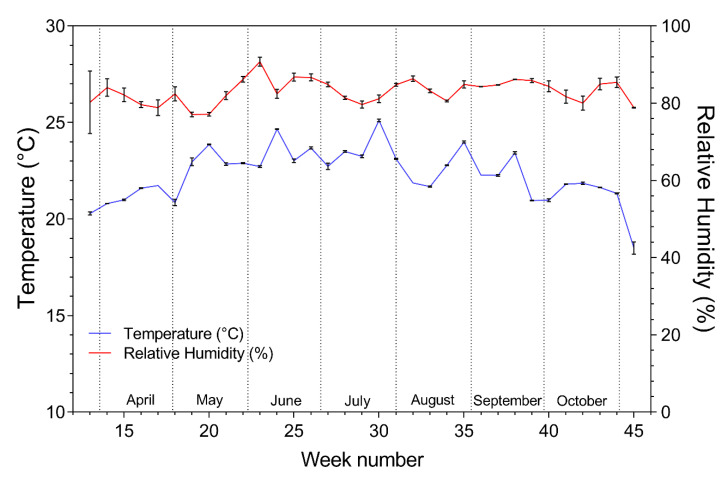
Weekly mean (± SD) temperature and relative humidity in the reference and diffuse bell pepper compartment.

The daily mean temperature in the bell pepper greenhouse compartments was 21.3 ± 0.10 °C in the diffuse and 21.3 ± 0.10 °C (mean ± SE) in the reference treatment during the total length of the trial. The daily mean relative humidity was and 80.6 ± 0.5% in the diffuse and 82.8 ± 0.4% (mean ± SE) in the reference treatment. The weekly mean temperature and relative humidity in the reference and diffuse compartment were equivalent during the total length of the trial, as indicated by the SD in Figure 2.

### 2.2. Greenhouse Light Transmission

**Figure 3:**
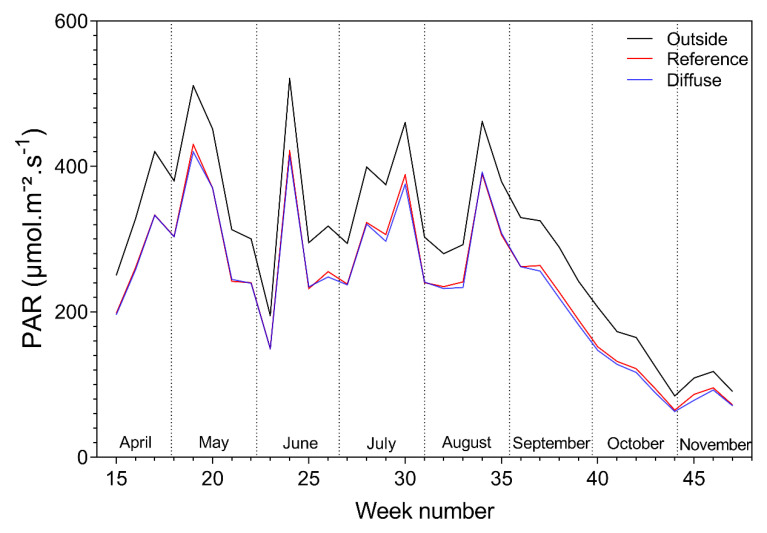
Weekly mean photosynthetic active radiation (PAR), outside and in the reference and diffuse tomato compartment.

**Figure 4:**
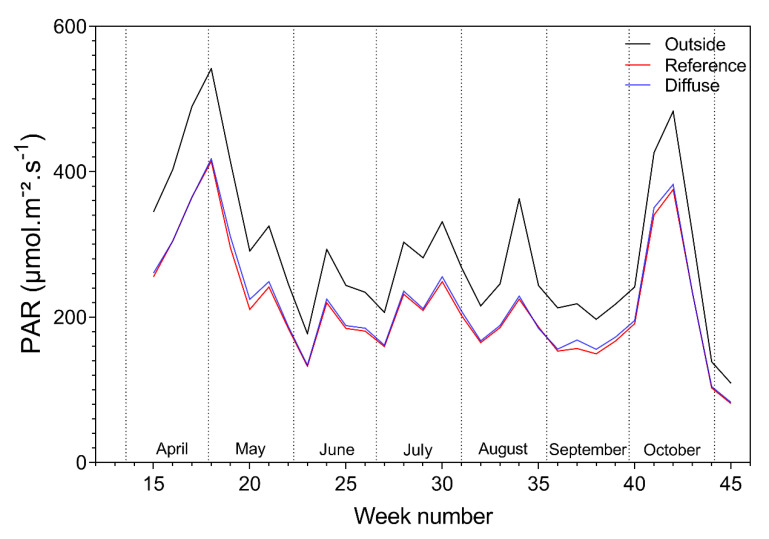
Weekly mean photosynthetic active radiation (PAR), outside and in the reference and diffuse bell pepper compartments.

During the total length of the trial, the PAR did not vary much between the reference and diffuse compartments for both tomato and bell pepper (Figure 3 and Figure 4). The measured PAR inside all compartments followed the same trend as the PAR measured outside; however, it was slightly lower in intensity. The PAR outside for bell pepper varies with the tomato one because only data is used where the thermal sunscreens are open.

The PAR light inside the greenhouse showed a linear relation with the PAR light outside the greenhouse for both the reference and diffuse glass for each month. The ANCOVA model showed significant differences in light transmission between glass types (F_2,4082_ = 90,718; *p* < 0.0001) and months (F_8,4082_ = 21.96; *p* < 0.0001). The yearly average light transmission was only 2.66% higher in the diffuse compartments compared to the reference (Table 1).

### 2.3. Vertical Light Distribution

**Figure 5:**
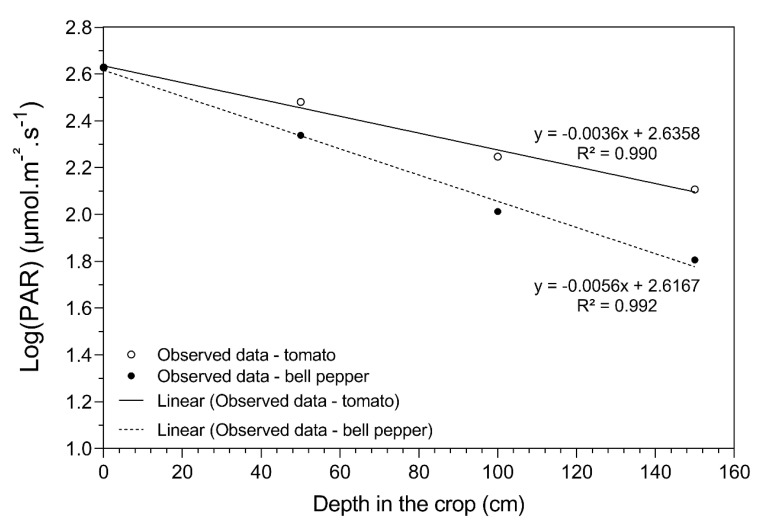
Example of a vertical light penetration measurement at four depths in a tomato and bell pepper crop with equal light intensity at the head of the plant and with the same type of glass. The vertical light distribution coefficient (VLDC) is represented by the slope of the linear regression fits through the four PAR measurements at different depths.

VLDC values were determined on the total dataset for tomato and bell pepper. An example of the calculation of the VLDC for the two crops is illustrated in Figure 5. At equal light intensities at the head of the plant and with the same type of glass, deeper light penetration was observed for tomato compared to bell pepper.

**Figure 6:**
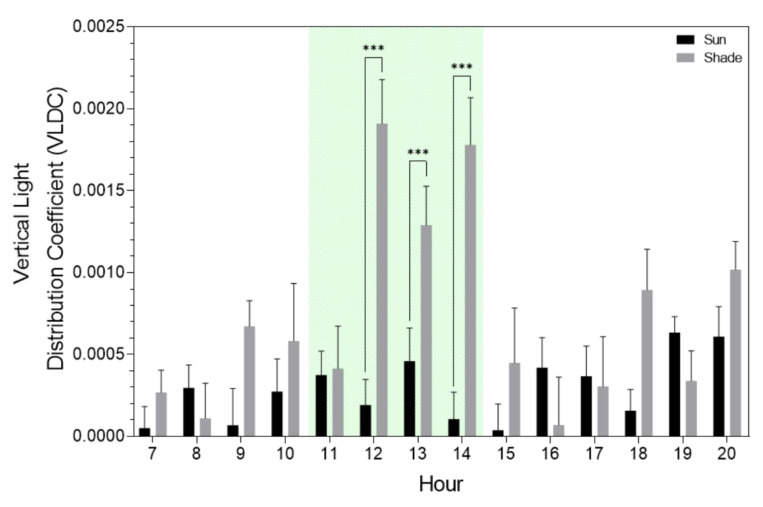
Hourly mean (+ SE) differences of the vertical light distribution coefficient (VLDC) between the reference and diffuse tomato compartment at the sun and shaded side of the rows. Stars indicate a significant difference (***, *p* < 0.0001) (two-Way ANOVA), green shading indicates data selection time points.

Further analysis of the VLDC only included data points between 11:00 and 14:00. This is the moment of the day that typically receives the most irradiation. Furthermore, for this time slot, we observed significant differences in the VLDC between the reference and diffuse compartment between the sun and shaded side of the row (Figure 6).

#### 2.3.1. Vertical Light Distribution for Tomato 

**Figure 7:**
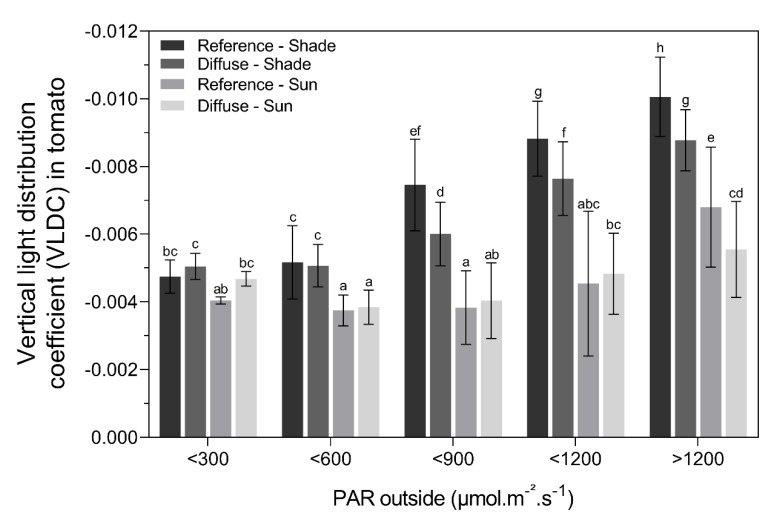
Mean (± SE) vertical light distribution coefficient per PAR light category in the reference and diffuse glass compartment at the sun and shade side of the tomato canopy. Different letters indicate significant differences (two-way ANOVA, post-hoc Tukey test).

On very dark days (PAR < 300 μmol.m^−2^s^−1^ between 11:00–14:00), the VLDC was equal for both tomato compartments with different glass types (reference vs. diffuse) and the sun/shade plant rows (Figure 7). When the light intensity increased, the VLDC on the shade sides decreased significantly compared to the sunny sides for both glass types. This implies a lower vertical light distribution on the shade side of the canopy. When PAR levels were higher than 600 μmol.m^−2^s^−1^, light penetrated deeper into the canopy on the shade side with diffuse glass compared to the reference. At the sun side of the plant rows, the effect of light intensity or diffuse/direct light on the VLDC appeared to be less relevant. Only on very sunny days (PAR > 1200 μmol.m^−2^s^−1^ between 11:00 and 14:00), the light penetrated deeper into the crop on the sun side with diffuse glass. 

#### 2.3.2. Vertical Light Distribution for Bell Pepper

**Figure 8:**
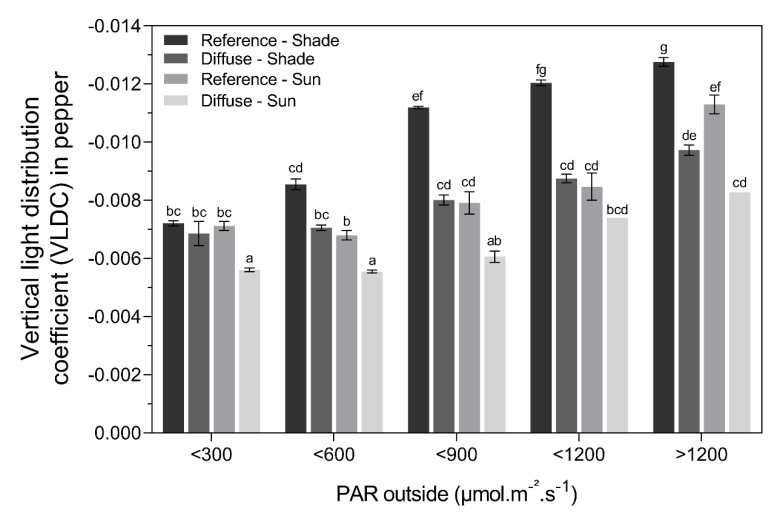
Mean (± SE) vertical light distribution coefficient per PAR light category in the reference and diffuse glass compartment at the sun and shade side for the bell pepper canopy. Different letters indicate significant differences (two-way ANOVA, post-hoc Tukey test).

The variation in VLDC values was more constant for bell pepper (Figure 8). On very dark days (PAR < 300 μmol.m^−2^s^−1^ between 11:00 and 14:00), the light penetrated deeper into the crop in the diffuse compartments at the sun side of the plant rows compared to the other treatments. At light intensities of 600 μmol.m^−2^s^−1^ and higher, the vertical light penetration was lower with reference glass compared to diffuse glass, for both the sun and the shade side of the plant rows. Light penetration was similar between the sun side in the reference treatment and the shade side in the diffuse treatment. Differences in VLDC between the sun and shade side in the diffuse compartment were small. 

### 2.4. Production and Fruit Weight 

We wondered if the differences in the vertical light distribution throughout the tomato and bell pepper canopy also resulted in differences in fruit yield. We also examined if the diffuse glass has an effect on tomato yield when grown at different stem densities.

#### 2.4.1. Tomato Yield

**Figure 9:**
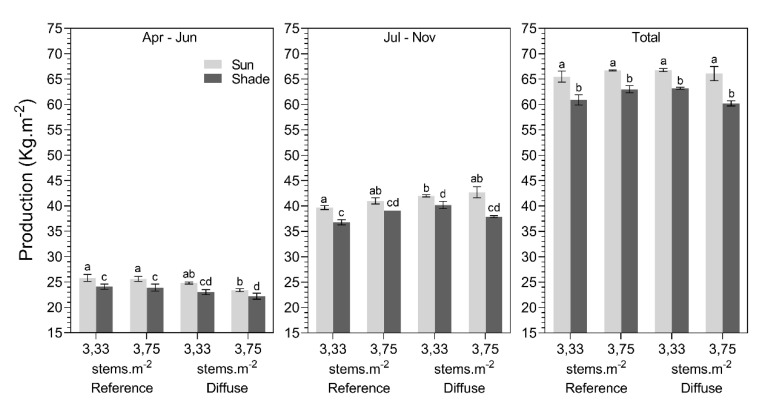
Average production (kg.m^−2^) for different stem densities (3.33 vs. 3.75 stems.m^−2^), glass types (reference glass vs. diffuse glass), and illumination position (sun vs. shaded side) during April–June, July–November, and the total cropping season. Different letters indicate significant differences per period (two-way ANOVA, pairwise Tukey test).

**Figure 10:**
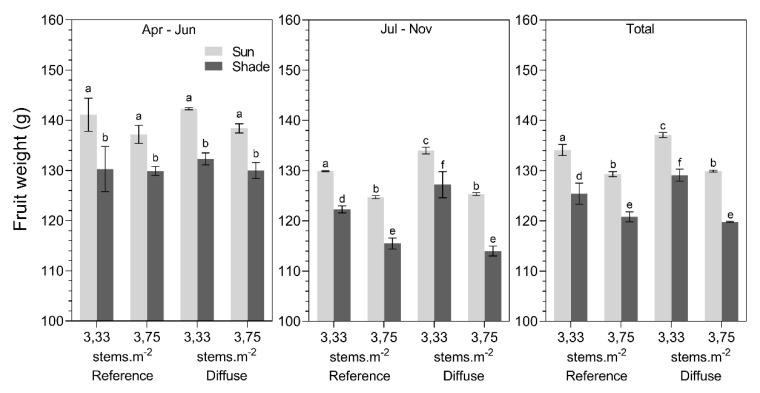
Average fruit weight (g) for different stem densities (3.33 vs. 3.75 stems.m^−2^), glass types (reference glass vs. diffuse glass), and illumination position (sun vs. shaded side) during April–June, July–November, and the total cropping season for tomato. Different letters indicate significant differences (two-way ANOVA, pairwise Tukey test).

Plant stems at the sun side of the plant rows had a significantly higher production (± 6%; Figure 9) and fruit weight (± 8%; Figure 10) compared to plant stems at the shade side. The total production (kg.m^−2^) of plants with the lowest stem density (3.33 stems.m^−2^) was ± 2.8% higher in the greenhouse with diffuse glass compared to the reference greenhouse, which was nearly significant (*p* = 0.052) (Figure 9). The glass type did not affect production numbers before summer (April–June); however, significant results were found during the period from July to November. During this period, fruit yield was 7.5% higher under diffuse glass compared to the reference for the lowest stem density (3.33 stems.m^−2^) (Figure 9). Increasing the stem density to 3.75 stems.m^−2^ did not increase the total production, nor the period during the July to November period, when comparing the reference with diffuse glass.

Overall, the mean fruit weight was significantly higher in the greenhouse with diffuse glass (around 3 g) compared to the reference glass (Figure 10). These differences in fruit weight were only observed in the period July–November and for the lower stem density (3.33 stems.m^−2^). During this period, fruits were 4–5 g heavier (Figure 10). Remarkably, the fruit weight of the plants cultivated at a higher stem density (3.75 stems.m^−2^) did not differ between the reference and diffuse treatments throughout the entire cropping period. On average, fruit weight was 5 g less in the reference and 8 g less in the diffuse compartment for the high stem density in comparison with the low stem density. 

#### 2.4.2. Bell Pepper Yield

**Figure 11:**
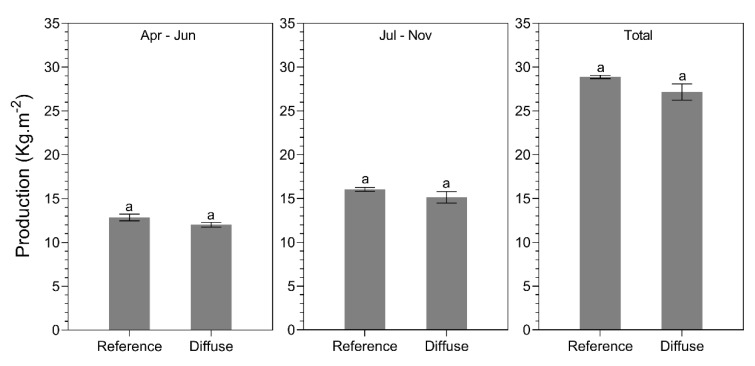
Average production (kg.m^−2^) for different glass types (reference glass vs. diffuse glass) during April–June, July–November, and the total cropping season for bell pepper. Different letters indicate significant differences (two-way ANOVA, pairwise Tukey test).

**Figure 12:**
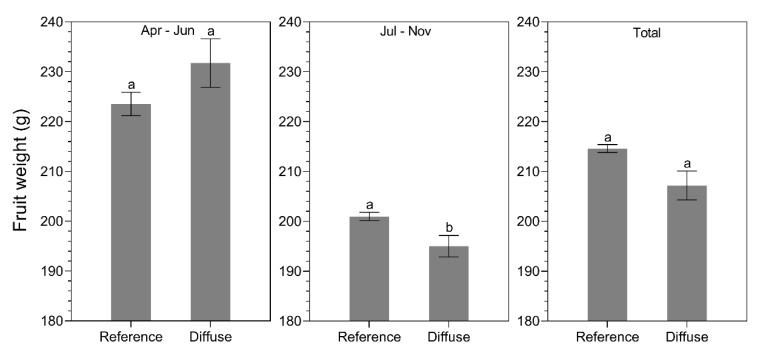
Average fruit weight (g) for different glass types (reference glass vs. diffuse glass) during April–June, July–November, and the total cropping season for bell pepper. Different letters indicate significant differences (two-way ANOVA, pairwise Tukey test).

The production (Figure 11) and fruit weight (Figure 12) of the sun and shade side of the plant row were not significantly different for bell pepper (*p* = 0.8). In contrast to tomato, there were no significant differences in production between the diffuse and reference treatment for all growing periods (Figure 11). However, a significant difference (*p* = 0.04) in fruit weight was observed only during July–November, with fruit being 8g heavier in the reference treatment compared to the diffuse treatment (Figure 12).

## 3. Discussion

The commercial cultivation of tomato and bell pepper in modern greenhouses can reach very high yields [23]. Often, greenhouse growers strive to further enhance their yield using novel technologies and innovative cropping systems. The use of diffuse glass is an emerging trend in the greenhouse production of many crops, although few studies have quantified the potential beneficial effects on yield. The advantage of diffuse glass is related to the ratio of natural occurring direct and diffuse light (i.e., the number of clouds) and is thus heavily season and location dependent [1,5,13]. On sunny clear days, more direct light reaches the greenhouse, creating more undesirable shading patterns. On cloudy days, natural light is already more diffuse compared to sunny clear days, which could benefit only the biomass production of certain shade-tolerant species [3]. According to Earles et al. [9], the cross-over point where diffuse light starts to benefit crop biomass production compared to direct light is 750 μmol.m^−2^s^−1^. Our results for tomato production confirm that diffuse light is only stimulating yield on sunny days (PAR > 600,750 μmol.m^−2^s^−1^). We observed a more homogeneous vertical light distribution with diffuse glass for both tomato and bell pepper when the total PAR was > 600 μmol.m^−2^s^−1^, suggesting that the light will penetrate deeper into the canopy. This resulted in an increase in production under diffuse glass for tomato (annual yield + 2.8%) but not for bell pepper. During the summer months, when there were more sunny days, the average yield was even higher (+ 7.5%) for tomato. Similar results were observed during sunny seasons for the commercial production of tomato and *Anthurium* under diffuse glass [12,22]. On sunny days, diffuse glass can enhance photosynthesis by about 7.2% [13]. A high-haze glass type even improves the diffuse/direct light ratio, also leading to larger fruits [12]. The reason why we did not observe an enhanced production under diffuse glass for bell pepper can be explained by (1) the denser canopy morphology of this crop, (2) the lack of a good leaf pruning strategy in bell pepper cultivation, and (3) the use of radiation screens on sunny days to prevent fruit sunburn damage [24]. Altogether, our results suggest that crop type and seasonal effects drastically influence the vertical light penetration, and will determine the light offset at which diffuse glass becomes beneficial for production. 

Besides crop type and seasonal effects, also canopy morphology, density, and orientation play an important role [4]. Our result showed that the beneficial effect of diffuse glass on tomato production on sunny days also has certain constraints. We only observed an enhanced production in tomato yield (+ 2.8%) and fruit weight (+ 4–5 g) for plants grown with the low stem density (3.33 stems.m^−2^) and not with the high stem density (3.7 stems.m^−2^). Thus, increasing the stem density under low-haze diffuse glass is not recommended. We can conclude that an increase in stem density will decrease the vertical light distribution throughout the canopy and therefore counteracts the positive effect of diffuse glass. However, under greenhouse conditions with normal float glass, a higher stem density can lead to higher yields per surface area for some tomato cultivars [25,26]. 

Another factor to consider is the orientation of the cultivation rows. In our case, the overall vertical light distribution at the shade side of the tomato plant rows was generally much lower compared to the sun side due to the east–west orientation of the greenhouse compartments, resulting in a significant production loss of 6% and a decreased fruit weight of 8% for the shaded side compared to the sunny side. We did not observe a beneficial effect of diffuse glass on tomato yield at the shade side of the plant rows, even though the VLDC was more pronounced at this shaded side under diffuse glass compared to the reference glass. At the sun side of the plant rows, diffuse glass only had a slightly better light penetration on the clearest days (PAR > 1200 μmol.m^−2^s^−1^). 

Altogether, our results show that shaded or denser crops, such as bell pepper or tomatoes grown at a high stem density or at the shaded side of the canopy, benefit less from the diffuse glass with low haze during sunny days, compared to more open crops. The total amount of light that reaches the bottom of a dense crop is much lower compared to a more open crop. Therefore, a lower photosynthetic activity of the lower leaves is expected [9,12,13]. The diffuse glass will only benefit the photosynthesis of the lower leaves if the outside light intensity is high enough and the canopy density low enough. Additionally, unwanted photoinhibition in the upper part of the canopy during days with high light intensities will negatively impact photosynthesis [5,9] and can eventually reduce yield under float glass, which can be overcome by using diffuse glass [13]. So, a careful selection of the crop type and the associated cultivation techniques (e.g., leaf pruning strategy, row orientation, stem density) will determine if the diffuse glass is beneficial for production or not.

## 4. Materials and Methods

### 4.1. Greenhouse Specifications

Two east–west-orientated greenhouse compartments of 500 m^2^ in the city of Hoogstraten, located in the Belgian province of Antwerp, were equipped with low-iron diffuse glass, including a double AR coating (Albarino, Saint-Gobain). Under lab conditions, the measured hemispheric light transmission of the glass was 91% with a low haze of 20 ± 10%. Two other identical and adjacent compartments were equipped with standard float glass (Saint-Gobain) with a hemispheric light transmission of 84% and no haze under lab conditions. Hereafter, these compartments are referred to as “diffuse” and “reference” treatments, respectively. One of each compartment, respectively one with reference float glass and one with diffuse glass, was used for the tomato trial whilst the other two compartments were used for the bell pepper trial. These 7-m-high greenhouse compartments, with a crop wire height at 4.2 m, were equipped with a gutter growing system (FormFlex/Metazet, The Netherlands), which were placed at a height of 0.7 m above ground with an inter-row spacing of 1.6 m for tomato and 1.36 m for bell pepper. Climate conditions were automatically logged in each compartment and registered using a Priva Electronic Measuring Box (Priva, The Netherlands). The average diurnal temperature was set to be 20/21 °C. CO_2_ levels were continuously measured and kept between 800 and 1000 ppm. Furthermore, the bell pepper compartments were additionally equipped with two thermal screens SLS 10 Ultra Plus (Svensson, Sweden) to prevent fruit sunburn during moments of high light intensities. These screens were closed when the light intensity exceeded 600–650 W.m^−2^.

### 4.2. Crop Specifications

The cluster tomato (*Solanum lycopersicon* L.), cultivar Foundation (Nunhems/BASF, The Netherlands), was sown on the 5th of November 2015 and planted on the 5th of January 2016 in both the diffuse and reference compartment. Plants were grafted on the rootstock Maxifort (De Ruiter, The Netherlands) and planted at 0.5-m intervals on Rockwool slab substrate (Grodan, The Netherlands), resulting in a density of 2.5 stems.m^−2^. To alter stem density, one additional auxiliary stem was retained per three stems in one plot and per two stems in a second plot seven weeks after planting. This resulted in final stem densities of respectively 3.33 and 3.75 stems.m^−2^. Plants were fertigated using drip irrigation and the frequency thereof was modulated based upon the radiation and plant age whilst taking 20–30% of the drain into account. The nutrient composition was adapted every two weeks to the plants' needs by analyzing the drain water. Fruits were harvested and documented one or two times per week from the 6th of April 2016 until the 14th of November 2016. Biological control agents *Macrolophus pygmaeus*, *Encarsia Formosa*, and *Phytoseiulus persimilis* were released according to the advice of the biological advisor (Biobest N.V., Belgium). However, no significant pests or pathogen outbreaks occurred during the experiment. 

The bell pepper plants (*Capsicum annuum* L.), cultivar Maduro (Enza Zaden, The Netherlands), were sown on the 21st of October 2015 and planted in the greenhouse on the 7th of December 2015. The plants were planted at 0.32-m intervals on Rockwool slab substrate (Cultilene, The Netherlands), resulting in a plant density of 2.37 plants.m^−2^. Each plant had three stems, 1 main and 2 auxiliary stems, which resulted in a stem density of 7.1 stems.m^−2^. Plants were fertigated using drip irrigation and the frequency thereof was modulated based upon the radiation and plant age whilst taking 20–30% of the drain into account. The nutrient composition was adapted every two weeks to the plants’ needs by analyzing the drain water. Fruits were harvested and documented once every week from the 23rd of March 2016 until the 2nd of November 2016. Biological control agents *Macrolophus pygmaeus*, *Eretmocerus eremicus*, and *Aphidius* spp. were released according to the advice of the biological advisor (Koppert B.V., The Netherlands). However, no significant pest or pathogen outbreaks occurred during the experiment. 

For both tomato and bell pepper, four individual production plots per greenhouse compartment were monitored during the entire experimental period. Each plot contained 15 and 30 consecutive stems for tomato and bell pepper, respectively, leading to a total of 60 and 120 biological replicates per crop. As a result of the row design and greenhouse orientation, half of the stems were located on the sunnier side of the row whilst the other half at the more shaded side.

### 4.3. Production and Fruit Weight

Production (kg.m^−2^) and fruit weight (g) were registered for each harvest moment. A two-way ANOVA with a pairwise post-hoc Tukey was used to check for significant differences between treatments for each crop separately. Because there can be a lot of variation in production between the sun and the shade side of plant rows, this variable was included in the statistical model. Statistics were carried out in R Studio 3.5.2. and all data were checked for normality.

### 4.4. Light Transmission through the Greenhouse Glass 

Light, more in particular the photosynthetic active radiation (PAR), was measured every five minutes using quantum PAR sensors (LI-190R-BNC-2 Quantum Sensor, LI-COR, Catec, The Netherlands) and is expressed in μmol m^−2^ s^−1^. To measure light transmission through the diffuse and reference glass, five PAR sensors were used. One PAR sensor was placed on top of the greenhouse to measure the total incoming solar PAR radiation, whilst other sensors were placed inside each greenhouse compartment, just above the crop wire, to measure the available PAR radiation at the level of the crops. Individual measurements for the two compartments with the same glass type were merged, leading to two light transmission replicates per glass type. PAR light outside and inside the greenhouse (from 11:00–14:00) were plotted against each other and a linear regression line through the origin was fitted for each month and treatment (R Studio 3.5.2), using an ANCOVA model with treatment and month as categorical variables. The slopes of these regression lines represent the proportion of light inside the greenhouse and thus the light transmission. 

### 4.5. Vertical Light Distribution through the Crop Canopy

The vertical light distribution throughout the crop canopy was measured simultaneously every five minutes using four quantum PAR sensors (LI-190R-BNC-2 Quantum Sensor, LI-COR, Catec, The Netherlands), stacked at four different heights within the canopy: 0 (head of the plant; above the canopy) and 0.5 m, 1.0 and 1.5 m below the canopy top. These measurements were done from the 1st of August 2016 until the 11th of September 2016 for tomato and from the 19th of August 2016 until the 23rd of September 2016 for bell pepper. PAR sensors were placed alternating on the sun and shade side of the production plots in all compartments for two to seven consecutive days. Each light measurement was log-transformed for data linearization, plotted against the height position, and a linear regression line was plotted. To take the accidental shading of individual PAR sensors by overhanging leaves into account, all regression lines with an R^2^ < 0.90 were removed. Additionally, for bell pepper, extra light penetration data points were removed when solar screens blocked the incoming light. 

The slope of each regression line was used to compare the vertical light distribution between compartments and between the sun and the shade side of the plant rows. Hereafter, this slope is named the vertical light distribution coefficient (VLDC). Here, a small VLDC means a poor vertical light distribution throughout the crop canopy due to more light attenuation. These VLDC values were categorized according to the amount of PAR light outside the greenhouse for both treatments and crops at the sun and shade sides (<300, <600, <900, <1200, >1200 μmol.m^−2^s^−1^). Only data between 11:00 and 14:00 were taken into account. This way, low PAR intensities could be related to cloudy skies and high PAR intensities with sunny clear skies. Because more natural diffuse light is present on a cloudy day and more direct light on sunny days, these PAR categories represent different ratios of diffuse and direct light. Statistical differences between VLDC values for the different treatments were analyzed using a two-way ANOVA with a pairwise post-hoc Tukey test. All statistics were carried out in R Studio 3.5.2.

## 5. Conclusions

Tomato cultivated in a more open canopy will produce more and larger fruit under low-haze diffuse glass combined with an AR coating, especially during sunny clear days. A denser tomato crop and a bell pepper crop do not benefit from low-haze diffuse glass. The reason is the limited vertical light distribution throughout a dense crop canopy, which counteracts the positive effect of diffuse glass on mainly the lower shaded leaves. The vertical light distribution depends on the crop type and cultivation techniques and should be optimized by, for example, altering the stem density, leaf pruning strategy, or row orientation. In bell pepper cultivation, the use of radiation screens to prevent sunburn eliminates the positive effects of low-haze diffuse glass, even during sunny clear skies. However, how these crops perform when grown using high-haze glass is yet to be elucidated.

## Figures and Tables

**Table 1:** Monthly light transmission (%) per glass type calculated as the slope of the linear regression line between PAR outside and inside the greenhouse compartments.

Month	Light Transmission (%)		
	Reference	Diffuse	Difference
Mar	83	85	2
Apr	79	82	3
May	76	79	3
Jun	76	79	3
Jul	80	83	3
Aug	81	83	2
Sep	83	86	3
Oct	79	81	2
Nov	75	78	3
Mean	79.11	81.78	2.66
